# Rapid progression and extensive lymph node metastases of papillary thyroid carcinoma in an HIV-positive patient: a Case Report

**DOI:** 10.3389/fmed.2025.1600307

**Published:** 2025-08-20

**Authors:** Ze Zheng, Xiang Chen

**Affiliations:** Department of Thyroid Breast Surgery, The Affiliated Yixing Hospital of Jiangsu University, Wuxi, China

**Keywords:** papillary thyroid carcinoma, HIV-positive, lymph node metastasis, immunosuppression, antiretroviral therapy

## Abstract

Human Immunodeficiency Virus (HIV)-induced immunosuppression represents a potential risk factor for tumorigenesis and cancer progression, though existing studies have not conclusively established the association between HIV infection and the proliferation/metastasis of papillary thyroid carcinoma (PTC). We present a rare case of a 26-year-old male patient who exhibited rapid cervical tumor enlargement with extensive high-burden lymphatic metastasis following HIV infection. Imaging examinations revealed a cystic-solid thyroid mass with multiple lymphadenopathies in bilateral cervical regions, mediastinum, and axillae. The patient initiated antiretroviral therapy (ART) upon HIV diagnosis and subsequently underwent surgical intervention followed by adjuvant iodine-131 therapy and Thyroid hormone suppression therapy. No recurrence was observed during the 15-month follow-up period. This report highlights a potential association between HIV infection and aggressive progression/metastatic potential in thyroid carcinoma, while highlighting the critical importance of personalized treatment strategies for optimizing clinical outcomes in HIV patients with concurrent PTC.

## 1 Introduction

Papillary thyroid carcinoma (PTC), the most prevalent thyroid malignancy, generally carries a favorable prognosis (1). However, the clinical outcome significantly deteriorates when accompanied by large-volume lymph node metastasis (defined as > 5 metastatic lymph nodes), posing substantial therapeutic challenges (2, 3). The association between HIV infection and thyroid cancer warrants attention. Previous studies indicated that the incidence of thyroid cancer among HIV-infected individuals ranges from ~4% to 8.5% (4, 5), with medullary thyroid cancer notably more frequent in this population than in the general population (6). Recent research has revealed that, compared with the general population, PTC in HIV-positive patients presents unique clinicopathological characteristics, specifically larger primary tumor size at diagnosis and a higher propensity for lymph node metastasis (7). This finding aligns with broader evidence suggesting that HIV infection may be associated with a more aggressive clinical course in certain malignancies (8). The HIV-induced immunosuppressed state is postulated to significantly enhance tumor immune evasion mechanisms. This impaired immune surveillance may not only promote tumorigenesis but could also facilitate more aggressive tumor progression and metastatic dissemination through various pathways (7–9).

Against this background of potential alterations in disease biology among HIV-positive individuals, we present a distinctive case highlighting these concerns. This case involves a young male PTC patient who, following HIV acquisition, experienced rapid neck tumor enlargement accompanied by extensive high-burden nodal metastases, suggesting a potential link between HIV infection and accelerated thyroid cancer development. This report details the aggressive clinical trajectory and successful multidisciplinary management to advance understanding of this complex interplay.

## 2 Case presentation

A 26-year-old male presented to Yixing People's Hospital on November 25, 2023, with a right anterior neck mass persisting for 1 year and progressively enlarging over the past 3 months. The patient first noticed a painless mass in the anterior neck region in November 2022, which was not accompanied by neuropathic compressive symptoms such as hoarseness or dysphagia. Since August 2023, the mass exhibited significant enlargement associated with respiratory compromise, prompting his visit to our institution.

## 3 Physical examination

**Figure 1:**
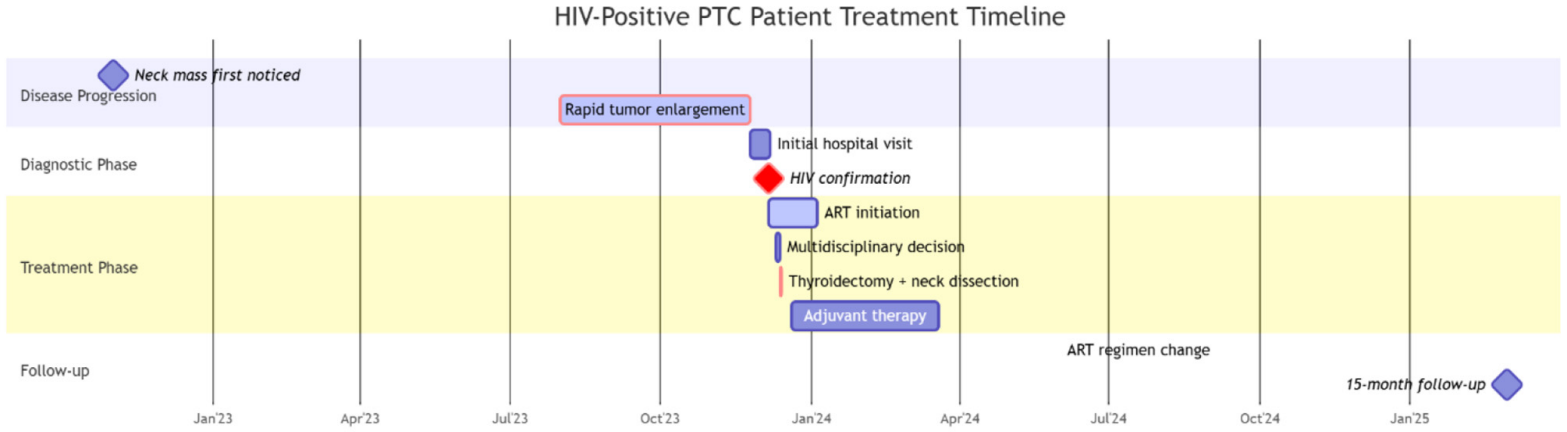
Timeline of the clinical events and treatment. PTC, Papillary thyroid carcinoma; ART, antiretroviral therapy.

Vital signs: temperature 36.8°C, heart rate 94 beats/min, respiratory rate 16 breaths/min, blood pressure 125/79 mmHg. Physical examination revealed a supple neck with mild leftward tracheal deviation. A 4 × 3 × 2 cm mass was palpated in the right anterior cervical region, demonstrating moderate consistency, mobility, and vertical mobility on deglutition, without tenderness. The overlying skin showed no erythema or edema. Enlarged lymph nodes were palpable in the lateral cervical regions. The patient's treatment timeline is presented in Figure 1.

## 4 Imaging and diagnostic findings

**Figure 2:**
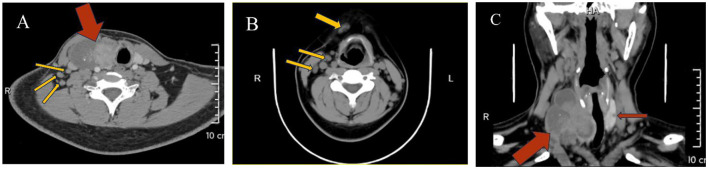
**(A)** A heterogeneous density lesion in the right thyroid lobe (red arrow) with tracheal compression and leftward displacement, accompanied by multiple enlarged lymph nodes in Level IV (yellow arrows); **(B)** Multiple enlarged lymph nodes in the anterior cervical region and Level III (yellow arrows); **(C)** A heterogeneous density lesion in the right thyroid lobe, a hypodense lesion in the left thyroid lobe (red arrows), with tracheal displacement to the left due to compression.

**Figure 3:**
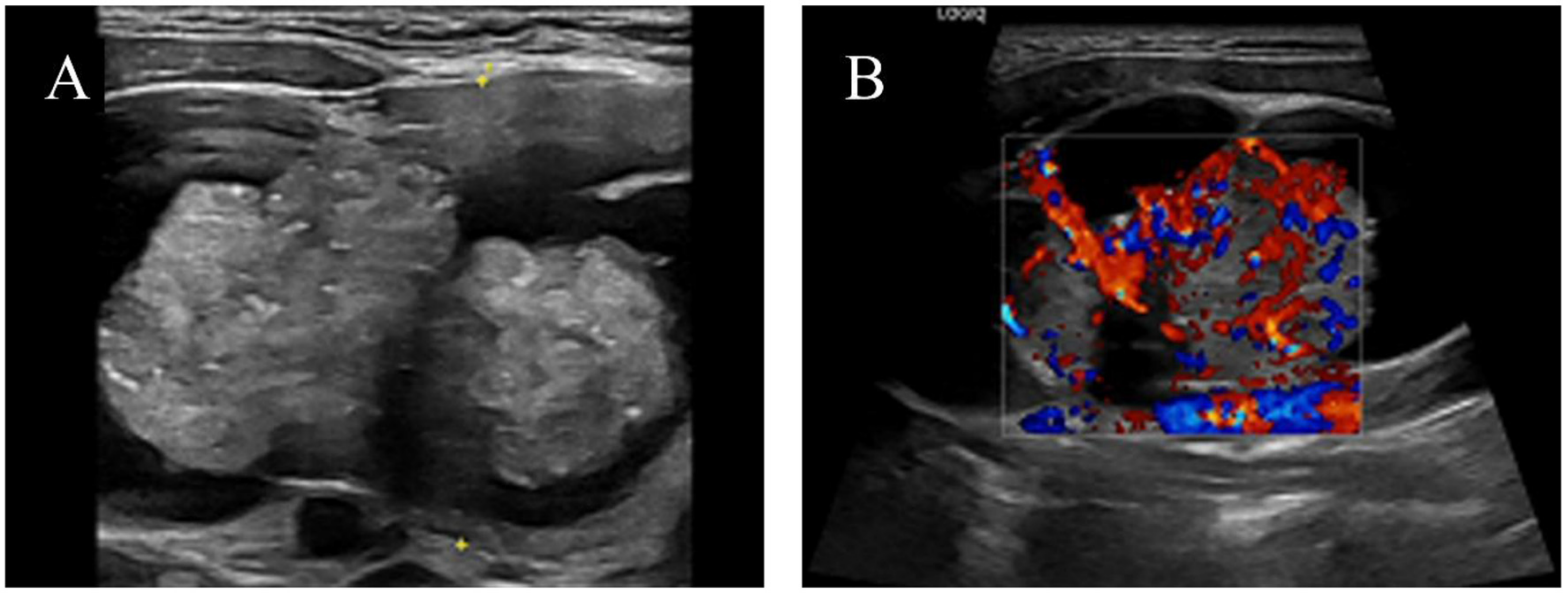
Color ultrasound images of the left thyroid lobe mass: **(A)** Cystic-solid mass; **(B)** Color Doppler flow imaging (CDFI) demonstrating abundant blood flow in the parenchymal portion.

HIV screening indicated pending confirmation of infection status. Thyroid function tests revealed elevated TSH (5.24 μIU/mL, normal range 0.27–4.2) and parathyroid hormone (78.60 pg/mL). Cervicothoracic CT (Figure 2) demonstrated a 74 × 43 mm heterogeneously enhancing mass with well-defined margins in the right thyroid lobe causing leftward tracheal deviation, accompanied by a 6 mm hypodense nodule in the left lobe. Extensive lymphadenopathy was observed in bilateral cervical chains (multiple nodes), axillary/infraclavicular regions, and mediastinum. Thyroid ultrasound (Figure 3) further characterized the right thyroid mass as a 98 × 74 × 38 mm mixed solid-cystic nodule with rich internal vascularity (C-TI-RADS 2), while the left lobe contained a 5.6 × 4.9 mm hypoechoic avascular nodule (C-TI-RADS 4). Cervical lymph nodes exhibited preserved hilar architecture, with the largest measuring 19 × 7 mm on the right and 14 × 9 mm on the left, demonstrating heterogeneous internal echoes but maintained morphological regularity.

## 5 Surgical treatment

Further history-taking revealed that the patient engaged in high-risk sexual behavior ~6 months ago, after which the patient self-administered an HIV antibody rapid test purchased online, reporting a negative result. Upon recommendation, the patient underwent confirmatory testing at the local CDC and was diagnosed with HIV infection on December 6, with laboratory results showing a CD4^+^ count of 237 cells/μL and an HIV viral load of 21,700 copies/ml. Immediate initiation of ART followed at our institution. Given the extensive lymphadenopathy with potential differential diagnoses including tuberculosis co-infection, the infectious disease team advised prioritized thyroidectomy with lymph node biopsy over empiric anti-tubercular therapy. The patient was readmitted on December 11 for surgical intervention, with the multidisciplinary team planning excisional biopsy of supraclavicular lymph nodes concurrent with thyroid surgery to establish definitive histopathological diagnosis.

**Figure 4:**
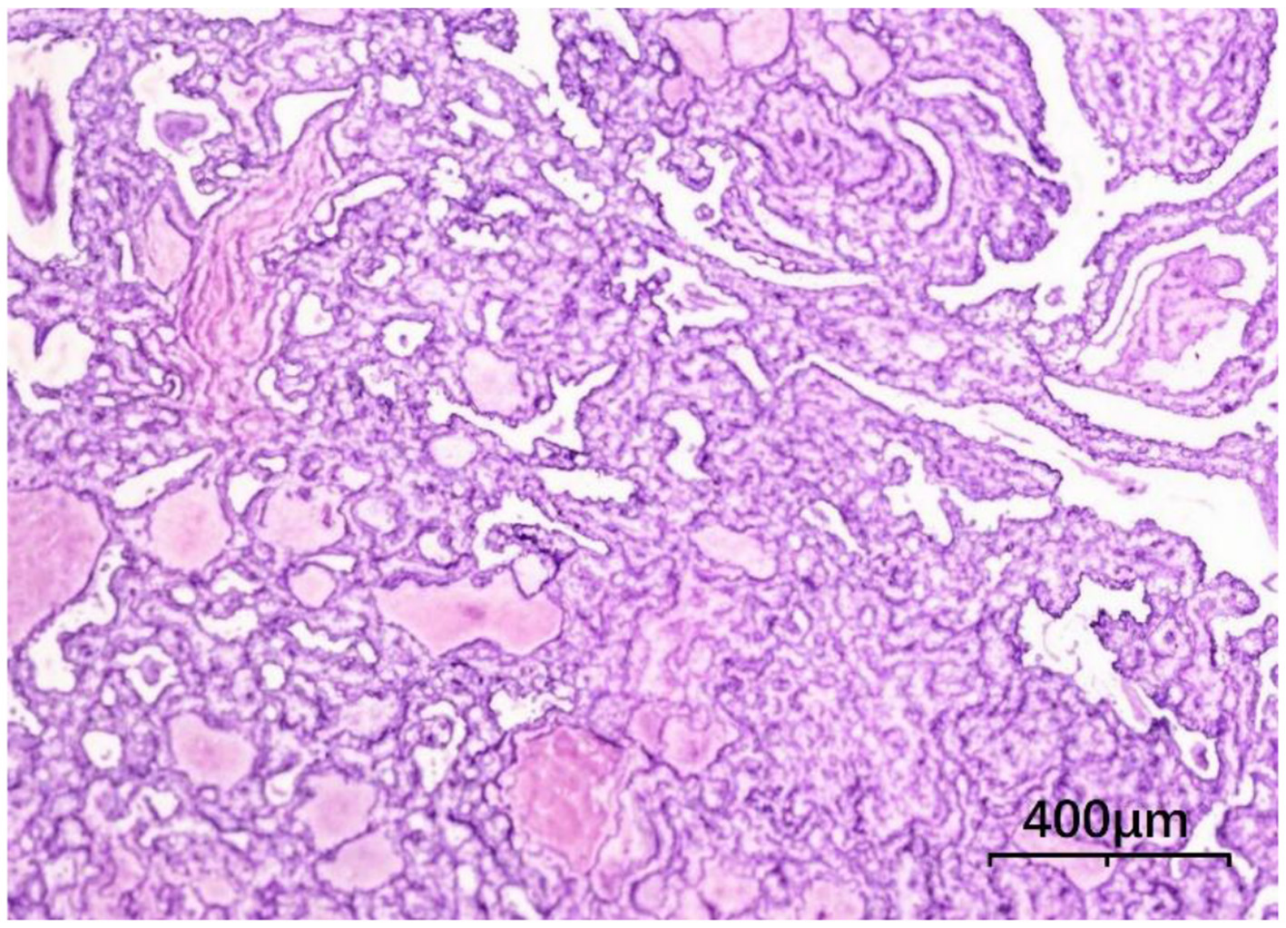
Thyroid histopathology, tumor cells are arranged in papillary patterns with crowding (HE × 100).

Following comprehensive preoperative evaluation to exclude surgical contraindications, the patient underwent total thyroidectomy with right-sided radical neck dissection (levels II, III, IV, VI) and left level VI functional lymph node dissection under general anesthesia on December 13. Histopathological examination revealed: Right lobe: Solid-cystic lesion with grayish-white solid components and dark red/amber cystic fluid, Left lobe: 4-mm grayish-white nodule. Final diagnosis: Bilateral papillary thyroid carcinoma (Figure 4), with metastatic involvement in 9/13 right level VI nodes and 6/11 right levels III-IV nodes. No metastasis detected in right level II (0/2) or left level VI nodes (0/6). Postoperative care included drain removal on day 4 and suture removal on day 7. The treatment plan comprised scheduled radioiodine (^131^I) therapy at an affiliated institution, lifelong levothyroxine sodium replacement, and continuation of ART with close monitoring of CD4^+^ counts and viral load.

## 6 Follow-up and outcome

Six months postoperatively, the ART regimen was transitioned to Biktarvy due to hepatotoxicity, with continued HIV viral suppression. Subsequent cervicothoracic CT imaging demonstrated no evidence of locoregional recurrence or residual disease. At the 15-month outpatient follow-up, the patient remains clinically stable and asymptomatic. Thyroid bed ultrasound revealed no sonographic evidence of residual/recurrent lesions in the surgical field, and serial thyroglobulin monitoring (TSH-stimulated Tg < 0.2 ng/mL) confirmed biochemical remission. Suppressive levothyroxine therapy was maintained (TSH ≤ 0.5 μIU/mL) per ATA recurrence-risk stratification and optimized ART; with a most recent CD4^+^ T lymphocyte count of 591/μL and an undetectable HIV-RNA, this approach demonstrates effective integration of oncological surveillance and immunodeficiency control.

## 7 Discussion

PTC is typically characterized by insidious onset and indolent biological behavior, often detected incidentally through neck palpation or imaging studies (1, 3). While generally associated with favorable prognosis, emerging evidence indicates that high-volume lymph node metastasis may significantly alter disease progression. Current literature reports a 3.4%−11.4% incidence of large-volume central compartment lymph node metastasis (> 5 nodes) in PTC (10–12). Notably, the present case exhibited an exceptionally high metastatic burden involving both central (9/13 nodes) and lateral cervical compartments (levels III-IV, 6/11 nodes). Such extensive lymphatic dissemination represents a clinically significant deviation from the metastatic patterns observed in immunocompetent PTC cohorts, may suggest a potential association between HIV-induced immunosuppression and accelerated metastatic spread.

In HIV-infected individuals, sustained high viral loads and progressive impairment of CD4^+^ T lymphocyte and B lymphocyte functions significantly enhance tumor immune escape mechanisms, potentially leading to more aggressive tumor biological behavior and promoting lymphatic dissemination of cancer cells (8, 9). In this case, the delayed diagnosis caused by a false-negative self-administered HIV antibody rapid test (likely during the acute/window phase) resulted in bilateral thyroid carcinoma with extensive high-burden lymph node metastasis at initial presentation, consistent with the clinical characteristic that HIV-associated malignancies are frequently diagnosed at advanced stages. The observed high metastatic burden and rapid progression may be closely associated with HIV-related immune surveillance defects, such as reduced NK cell activity. Studies (9, 13) demonstrate that HIV induces a chronic inflammatory state (e.g., elevated IL-8 levels) to enhance tumor cell migratory capacity, thereby facilitating lymph node metastasis. Furthermore, Liu et al. (7) proposed that HIV infection may impair hypothalamic-pituitary axis function, causing abnormally elevated TSH secretion that promotes PTC proliferation. The rapid tumor enlargement and concurrent TSH elevation in this case suggest synergistic acceleration of tumor progression through these mechanisms. Notably, while 15.0%-53.8% of thyroid nodules in the general population exhibit mixed cystic-solid features (17.6% malignant), cystic components typically originate from intratumoral hemorrhage/necrosis or follicular dilation (14, 15). The dark-red cystic fluid observed in the right lobe tumor section indicates old hemorrhage. In such lesions, solid cancerous foci may be obscured by cystic areas, leading to potential misclassification as benign on ultrasound (e.g., C-TI-RADS category 2 in this case), underscoring the necessity of combining imaging with biopsy for accurate diagnosis (15).

Lymphatic metastasis of thyroid carcinoma typically follows a sequential dissemination pattern, progressing from the ipsilateral central compartment (level VI) to lateral cervical regions (levels II-IV) or mediastinal nodes, while axillary and infraclavicular nodal involvement remains exceptionally rare (3). In this case, postoperative follow-up revealed non-metastatic mediastinal, axillary, and infraclavicular lymphadenopathy, potentially associated with HIV-induced immune dysregulation causing reactive hyperplasia, though differential diagnoses including Mycobacterium tuberculosis and fungal opportunistic infections require exclusion. Current preoperative nodal evaluation predominantly relies on ultrasonography and CT, yet their sensitivity limitations persist: ultrasonography demonstrates 73% sensitivity (95% CI, 64%−80%) for central compartment nodes, while CT achieves 77% sensitivity (95% CI, 67%-85%) for lateral cervical nodes (16). Generalized lymphadenopathy in HIV patients necessitates differentiation among HIV-associated lymphadenopathy, opportunistic infections, and metastatic disease (17). Thus, precise preoperative nodal assessment is critical for surgical planning. This case employed intraoperative frozen-section analysis and final histopathology to delineate metastatic spread and exclude non-neoplastic nodal pathology, providing pivotal guidance for therapeutic decisions. Furthermore, cervical lymph node metastasis severity correlates strongly with prognosis, serving as a key parameter in thyroid cancer risk stratification. Per ATA guidelines (3), large-volume nodal metastasis constitutes an intermediate-high risk factor for recurrence (7%−21%), with elevated distant metastasis potential. While HIV-specific nodal metastatic patterns remain incompletely characterized, the bilateral thyroid carcinoma with multi-compartment high-burden metastasis observed in this case suggests that HIV-mediated compromised immune surveillance mechanisms may accelerate tumor dissemination (18).

Relevant guidelines recommend that tumor diagnosis and treatment must not be compromised by concurrent HIV infection, requiring standardized antitumor therapy, along with early initiation of ART and careful management of potential drug-drug interactions between antiretroviral and antitumor agents (19). In patients with intermediate-to-high risk stratified papillary thyroid carcinoma, radioactive iodine therapy may synergistically cause bone marrow suppression with ART drugs. Through regimen optimization and close monitoring, the interaction risk should be reduced, thus ensuring safe and effective thyroid cancer treatment for people living with HIV.

## 8 Conclusion

In conclusion, HIV infection might be associated with heightened PTC aggressiveness in the context of immune disruption. Early ART initiation combined with oncological interventions (surgery, radioiodine) and vigilant surveillance for opportunistic infections are critical in this population. Multidisciplinary management optimizes outcomes, emphasizing the need for tailored protocols in HIV-associated thyroid malignancies.

## Data Availability

The original contributions presented in the study are included in the article/supplementary material, further inquiries can be directed to the corresponding author.
